# The Structure of the Brachial Plexus of the Djungarian Hamster (*Phodopus sungorus*)

**DOI:** 10.1007/s11259-021-09877-1

**Published:** 2022-01-04

**Authors:** Arkadiusz Grzeczka, Maciej Zdun

**Affiliations:** Department of Basic and Preclinical Sciences, Nicolaus Copernicus University in Torun, Lwowska 1, 87-100 Toruń, Poland

**Keywords:** Anatomy, Innervation, Neurology, Rodent

## Abstract

Hamsters are often chosen as companion animals but are also a group of animals frequently subjected to laboratory tests. As there are no scientific publications providing information on the anatomical architecture of the brachial plexus of the Djungarian hamster, this study analyses the structure of this part of the nervous system of this species. It is important to know the details of this structure not only for cognitive reasons, but also due to the increasing clinical significance of rodents, which are often used in scientific research. The study was conducted on 55 specimens. Like in humans, the brachial plexus of the Djungarian hamster has three trunks. The following individual nerves innervating the thoracic limb of the Djungarian hamster: the radial nerve, median nerve, ulnar nerve, musculocutaneous nerve, axillary nerve, suprascapular nerve, thoracodorsal nerve, cranial pectoral nerves, caudal pectoral nerve, lateral thoracic nerve, long thoracic nerve, and subscapular nerves. Similarly to other mammals of this order, the brachial plexus of the Djungarian hamster ranges widely (C5-T1). However, its nerves are formed from different ventral branches of the spinal nerves than in other mammals.

## Introduction

The Djungarian hamster is a species belonging to the Rodentia order and Cricetidae family (in the superfamily Muroidea). It is native to Siberia as well as semi-deserts in Kazakhstan (Steinlechner 1998). It is often used in laboratory investigations to study the influence of the photoperiod length on living organisms, as the colour of this species’ coat changes in response to the length of the day (Hoffman 1978). In summer the Djungarian hamster is black and brown, with a black stripe running along its back. In winter it becomes almost completely white, with a grey stripe. This trait is a determinant distinguishing the Djungarian hamster from the Campbell’s dwarf hamster (*Phodopus Campbelli*), which is a species of similar size and colour, but it does not change its hair colour for winter (Steinlechner 1998). The Djungarian hamster is also frequently chosen as a companion animal. The usefulness of researching the anatomy of the brachial plexus as well as the peripheral nerves originating from this plexus in companion animals is demonstrated by studies on common mammalian thoracic limb disorders (Thatte et al. 2013; Menchetti 2020; Anderson et al. 1970). As there are no scientific publications providing information on the anatomical architecture of the brachial plexus of the Djungarian hamster, this study analyses the structure of this part of the nervous system of this species. Such studies have been conducted on a number of rodent representatives. Most of the described species belong to the Porcupine suborder (Hystricognathi): capybara (*Hydrochaeris hydrochaeris*), African crested porcupine (*Hystrix cristata*), Spix's yellow-toothed cavy (*Galea spixii*), rock cavy (*Kerodon rupestris*) as well as nutria (*Myocastor coypus*), and small chinchilla (*Chinchilla lanigera*) (Araujo et al. 2016; Aydin 2003; Cevik-Demirkan et al. 2007; Fioretto et al. 2003; Guimaraes et al. 2013; Santana et al. 2003). However, representatives of the Myodonta suborder are also the subjects of scientific studies focused on brachial plexus analysis. The majority of brachial plexus related studies on Myodonta have been conducted on members of species belonging to the Muroidea superfamily. Species described from this group are, for example, the Mongolian gerbil (*Meriones unguiculatus*), mole-rats (*Spalax leucodon*), albino rat (*Mus norvegicus albinus*) (Araujo et al. 2018; Aydin and Karan 2012; Greene 1963). Studies devoted to this family are particularly relevant due to the similarity of species from Myodonta to human. It is important to know the details of this structure not only for cognitive reasons, but also due to the increasing clinical significance of rodents, which are often used in scientific research (Table 1).

**Table 1:** The results of our study against information on the brachial plexus of other rodents

Species	Nerves												Reference
	Suprascapular	Subscapular	Axillary	Cranial pectoral	Musculo-cutaneus	Radial	Thoracodorsal	Median	Ulnar	Cudal pectoral	Long thoracic	Lateral thoracic	
*Phodopus sungorus*	C5(C4-C5)	C5-C6	C5-C7	C6-C7	C5-C7	C7-T1	C6-T1	C6-T1	C8-T1	C7-T1	C7-C8	C8-T1	Our study
*Meriones unguiculatus*	C4-C6	C5-C6	C5-C7	C5-C6	C5-C7	C7-T1	C7-T1	C7-T1	C7-T1	C7-T1	C7-T1	C7-T1	Araujo et al. 2018
*Hydrochaeris hydrochaeris*	C4-C7	C5-C7	C5-C8	-	C6-T1	C6-T1	C6-T1	C6-T1	C6-T1	-	C6-C8	C7-T1	Fioretto et al. 2003
*Myocastor coypus*	C5-C6	C5-C7	C6-C7	C7	C6-C7	C6-C7	C7	C7-T1	C8-T1	C8-T1	C6-T1	-	Taketani 2017
*Galea spixii*	C6-C7	C6-C7	C6-C7	C6-C7	C7-C8	C8-T2	C8-T2	C7-T2	C7-T2	C7-T2	C7-C8	C8-T2	Araujo et al. 2016
*Agouti paca*	C5-C7	C6	C6-C7	C5-C8	C6-C8	C7-T1	C8-T2	C8-T1	C8-T1	C8-T1	C7-C8	C8-T2	Scavone et al. 2008
*Chinchilla lanigera*	C6	C6-C7	C6-C7	C7	C6-C7	C7-T1	C8	C7-T1	C7-T1	C8-T1	C7-C8	C8-T1	Gamba et al. 2007

## Material and methods

### Animals

The study was conducted on adult 55 specimens of the Djungarian hamster (25 males and 30 females). The Djungarian hamster carcasses were obtained from veterinary clinics. The animals had been euthanised [with xylazine 10 mg/kg (intramuscular; i.m.), ketamine 90 mg/kg (i.m.), and pentobarbital 100 mg/kg (intravenous; i.v.)] for other medical reasons than neurological disease.

### Methods

The research material was placed in a 10% formaldehyde solution for 72 hours. The examination was based on macroscopic preparation with magnifying equipment. In order to visualise the nerves, the sternum was cut, open and the contents of the thoracic cage cavity were removed. After the nerves had been separated from the muscles of the trunk and pectoral limb, swabs soaked with a 3% hydrogen peroxide solution were used to better visualise the nervous tissue and facilitate further preparation. The names of anatomical structures were standardised according to the Nomina Anatomica Veterinaria (International Committee on Veterinary Gross Anatomical Nomenclature 2017).

### Results

**Fig. 1:**
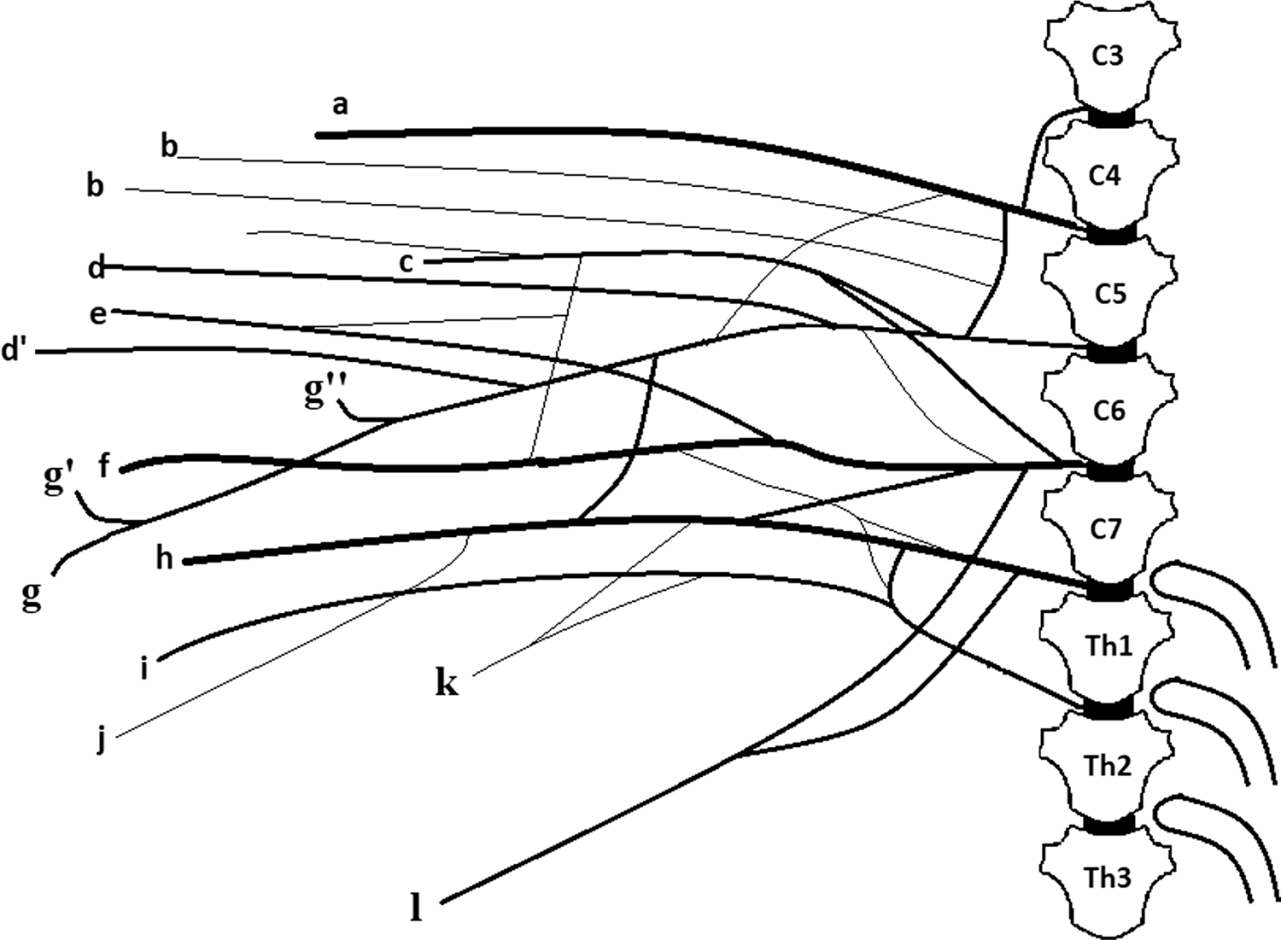
Diagram of the brachial plexus of the Djungarian hamster with a range of C4-T1. **a**. Suprascapular nerve **b**. Subscapular nerve **c**. Axillary nerve **d**. Cranial pectoral nerve **d’** Cranial pectoral nerve **e** Thoracodorsal nerve **f** Radial nerve **g**. Musculocutaneous nerve **g’** Proximal muscular branch **g”** Distal muscular branch **h** Median nerve **i** Ulnar nerve **j** Caudal pectoral nerve **k** Lateral thoracic nerve **l** Long thoracic nerve

**Fig. 2:**
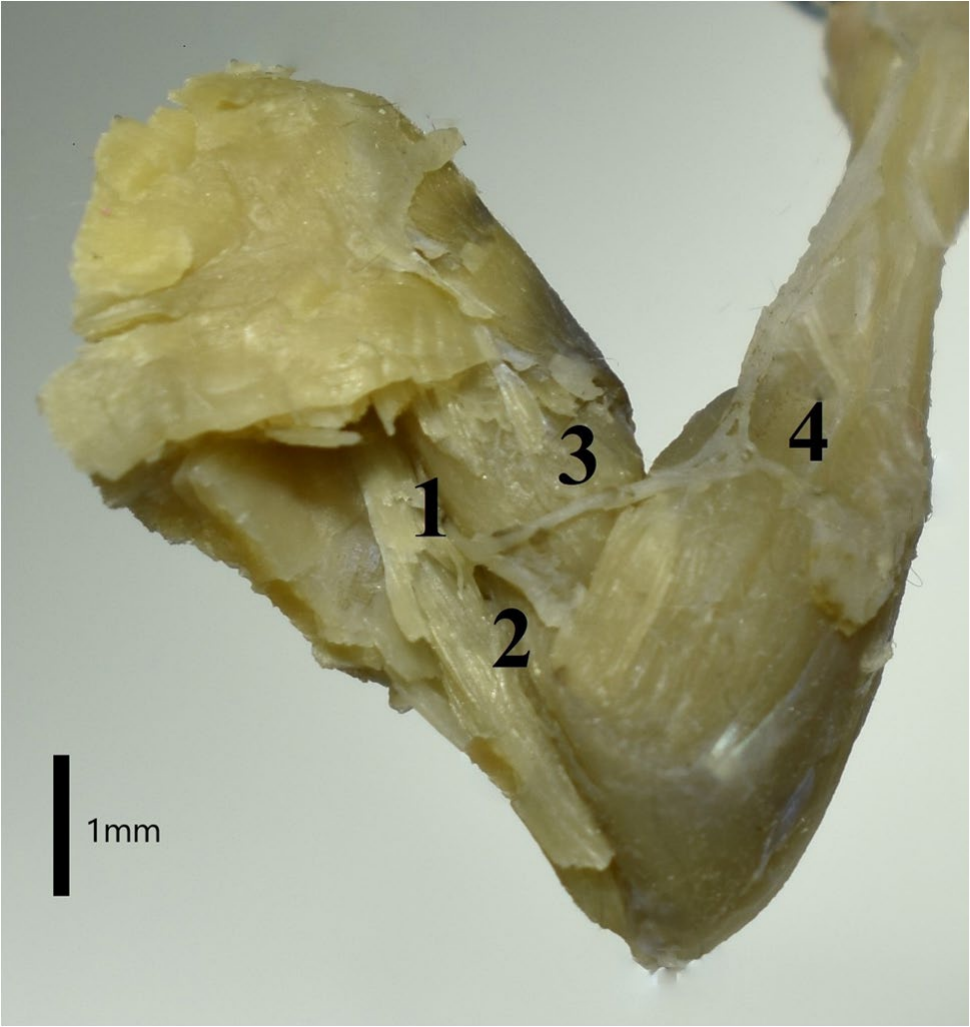
The course of the radial nerve on the lateral side of the right thoracic limb of the Djungarian hamster. The long head and lateral head of the triceps muscle were removed. 1. Radial nerve 2. Superficial branch of the radial nerve 3. Deep branch of the radial nerve 4. Lateral forearm cutaneous nerve

**Fig. 3:**
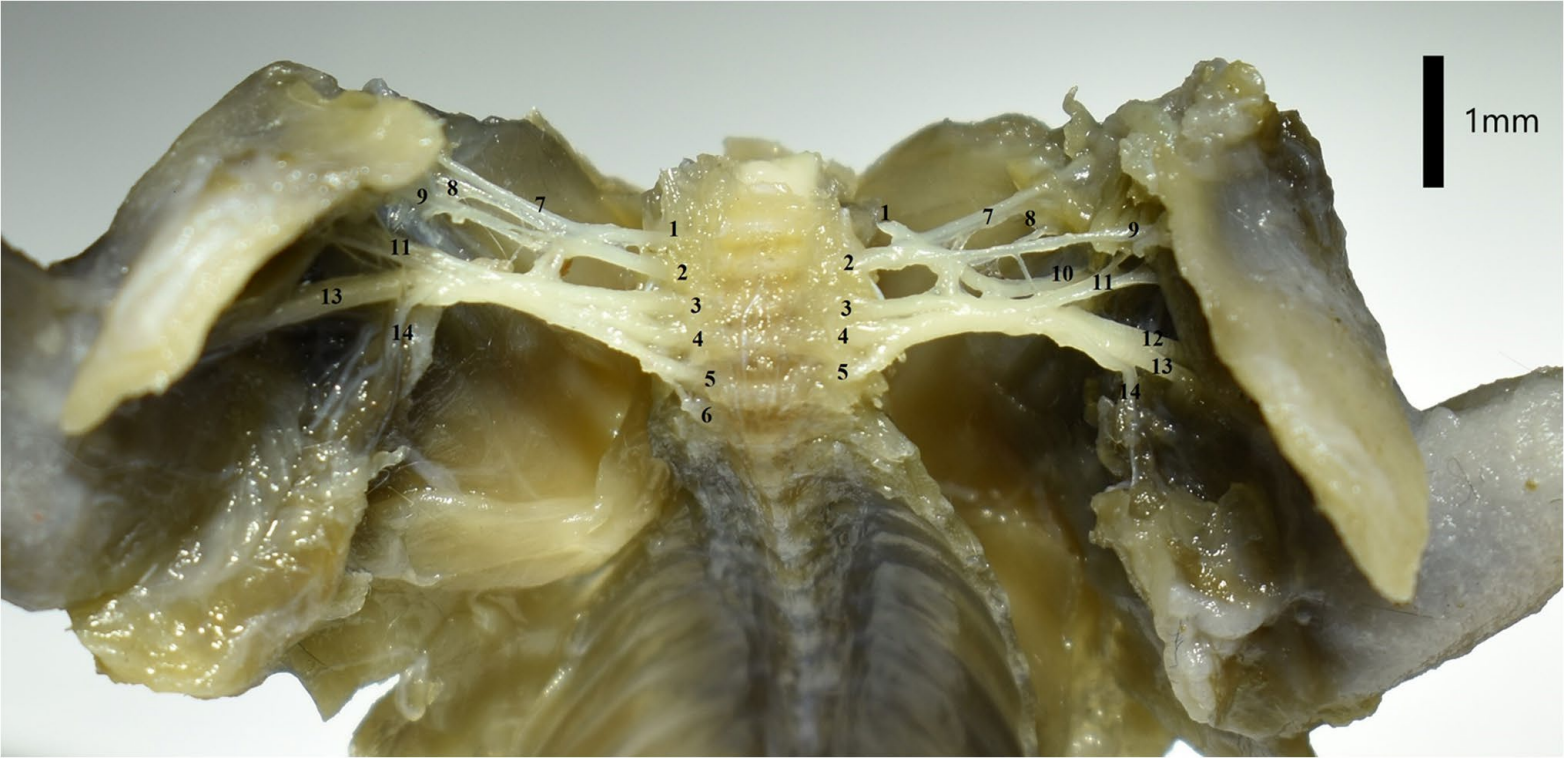
Ventral view of the brachial plexus of the Djungarian hamster. The plexus is C5-T1 on the left and C5-T2 on the right. The nerves shown in the photograph are: 1. C5 2. C6 3. C7 4. C8 5. T1 6. T2 7. Suprascapular nerve 8. Subscapular nerve 9. Cranial pectoral nerve 10. Axillary nerve 11. Musculocutaneous nerve 12. Radial nerve 13. Median and ulnar nerve 14. Long thoracic and lateral thoracic nerve

**Fig. 4:**
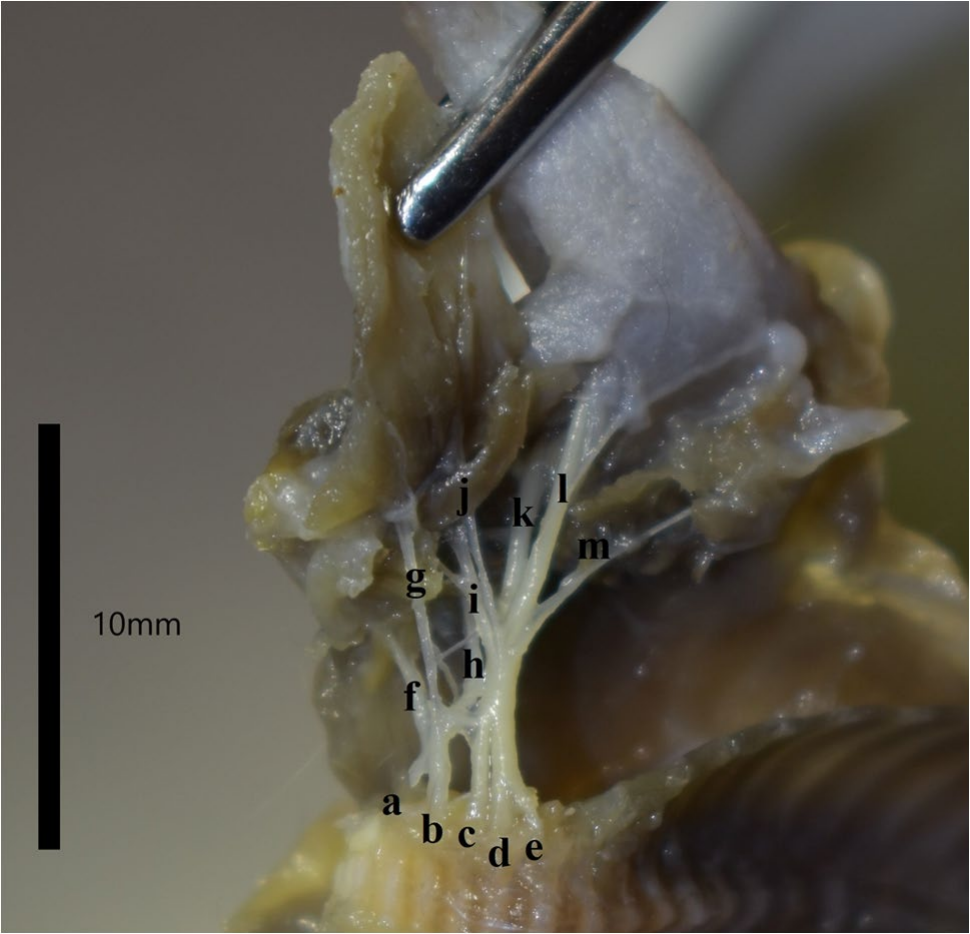
The brachial plexus of the Djungarian hamster in the left axillary fossa with a range of C5-T1. The tweezers deflect the pectoral muscle layer to visualise the plexus. The nerves shown in the photograph are: **a**. C5 **b**. C6 **c**. C7 **d**. C8 **e**. T1 **f**. Suprascapular nerve **g**. Cranial pectoral nerve **h**. Axillary nerve **i**. Musculocutaneous nerve **j**. Caudal pectoral nerve **k**. Radial nerve **l**. Median and ulnar nerve **m**. Long thoracic and lateral thoracic nerve

**Fig. 5:**
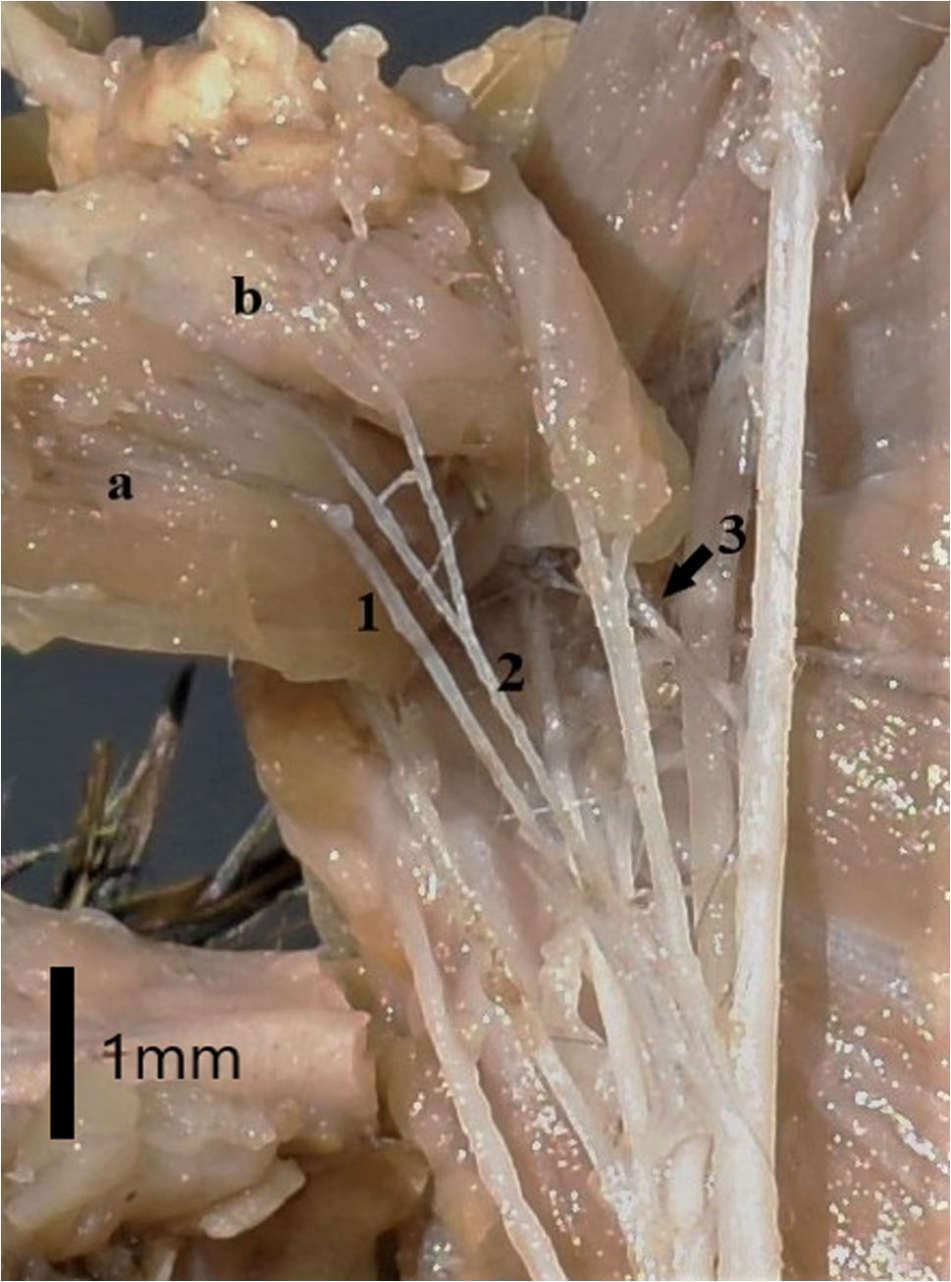
Range of innervation of the cranial pectoral nerves and caudal pectoral nerve in the Djunagrian hamster on the left side of the body. The nerves and muscles shown in the photograph are:1. Cranial pectoral nerve 2. Cranial pectoral nerve 3. Caudal pertoral nerve **a**. Superficial pectoral muscle **b**. Deep pectoral muscle

The brachial plexus of the Djungarian hamster was formed by ventral branches C4, C5, C6, C7, C8, T1, T2. The plexus was usually formed by branches C5-T1 (74%), or, much less frequently, by branches C4-T1 (22,8%). Rarely, a branch from T2 was attached to the plexus (3,2%) (Fig. 1). The network of ventral branches was formed by three trunks: the cranial trunk formed by branches C4-C7 (C4 via C5), the middle trunk formed by branch C7, and the caudal trunk formed by C8, T1 and T2 (T2 via T1). The following individual nerves innervating the thoracic limb of the Djungarian hamster spread from these trunks: the radial nerve (*n. radialis*), median nerve *(n. medianus*), ulnar nerve (*n. ulnaris*), musculocutaneous nerve (*n. musculocutaneus*), axillary nerve (*n. axillaris*), and suprascapular nerve (*n. suprascapularis*). Apart from these, other nerves spread from the plexus, i.e. the thoracodorsal nerve (*n. thoracodorsalis*), cranial pectoral nerves (*nn. pectorales craniales*), caudal pectoral nerve (*n. pectoralis caudalis*), lateral thoracic nerve (*n. thoracicus lateralis*), long thoracic nerve (*n. thoracicus longus*), and subscapular nerves (*nn. subscapulares*). The radial nerve is the largest nerve of the brachial plexus. It is mainly made of branches C7-T1. Near the middle of the humerus it goes between the medial head and the long head of the triceps muscle of the forearm. Next, there is a muscle branch for the tensor muscle of the antebrachial fascia and a branch for the medial head of the triceps muscle of the forearm, which is subdivided into a ramus for the rest of the head. There is also a ramus for the distal part of the brachial muscle in this area. Then, the radial nerve passes to the other side of the bone, under the lateral head of the triceps muscle of the forearm, where it splits into two ramus – one for the lateral head of the triceps and the other, which is an extension of the radial nerve. It runs along the cephalic vein in the forearm and innervates the extensor muscles of the wrist and digits with a deep branch, which diverges before crossing the elbow joint. It also innervates the forearm rotator muscles. The radial nerve also has a superficial branch, which extends towards the distal part of the limb. The superficial branch completes the sensory innervation of the forearm with the lateral cutaneous antebrachial nerve (Fig. 2). The main stem of the nerve branches into the common dorsal nerves of digits I-III at the metacarpus level. The median nerve is mainly formed by branches C6-T1. Up to a height of three quarters of the distal part of the humerus the median nerve is connected to the ulnar nerve by the connective tissue sheath (Fig. 3). The median nerve also receives a connecting branch from the musculocutaneous nerve. The branch is formed right behind the cranial. Before the elbow joint, the median nerve contacts the supracondylar foramen where it passes through. After crossing the joint the median nerve branches into the muscle ramus for: the radial flexor muscle of the carpus, the radial and brachial head of the flexor profundus muscle and the pronator teres muscle. After passing the wrist it goes towards the first digit on the palm side. The main stem located at the 1^st^ digit branches into the common palmar nerve of the 1^st^ digit, which splits into the axial proper palmar nerve of the 1^st^ digit and the abaxial proper palmar nerve of the 2^nd^ digit. Next, at the metacarpus level, the main stem branches into the common palmar nerves of the 2^nd^ and 3^rd^ digits. The common palmar nerve of the 2^nd^ digit splits into the axial proper palmar nerve of the 2^nd^ digit and the abaxial proper palmar nerve of the 3^rd^ digit. The common palmar nerve of the 3^rd^ digit branches into the axial proper palmar nerve of the 3^rd^ digit and the abaxial proper palmar nerve of the 4^th^ digit. The ulnar nerve consists of nerves C8 and T1, and it can attach to T2 via T1 (it was on the right side in one female). After splitting from the median nerve it branches into the caudal cutaneous antebrachial nerve and goes towards the medial surface of the elbow joint. Before entering the forearm it branches into a ramus to the anconeus muscle. Behind the elbow it innervates the forearm flexors (superficial digital flexor, flexor carpi ulnaris, ulnar head of the deep digital flexor muscle and brachial head). At the wrist level it branches into the dorsal and palmar branches. The dorsal branch first splits into the abaxial dorsal nerve of the 5^th^ digit and then the common dorsal nerve of the 4^th^ digit, which splits into the proper dorsal nerves of the 4^th^ and 5^th^ digits. The palmar branch innervates the same digits on the palmar side. The musculocutaneous nerve consists of branches C5-C7. Its stronger branch comes from C6 (71%) or C7 (29%). This nerve forms a branch which connects it to the median nerve. When it reaches the proximal attachment of the biceps, it branches into the proximal muscle ramus, which serves the biceps brachii. There are also thin secondary ramus branching from it to the coracobrachialis muscle and the brachial muscle near the proximal attachment of this muscle. The main trunk of the nerve runs under the biceps brachii on the bone and splits into another muscle branch near the distal attachment of the biceps brachii. Below the elbow joint it becomes the medial antebrachial cutaneous nerve. The axillary nerve is formed by branches C5-C7. The major branch forming the nerve can be C7 (64%) or C6 (36%). First, the axillary nerve fibres branch into one ramus to the subscapular muscle, innervating it in its caudal part, and another ramus to the teres major muscle. Then, behind the connection with the radial nerve, the fibres may branch into a very thin ramus to the latissimus dorsi muscle (it was on the right side of the body in one male) at the point where this muscle contacts the teres major muscle. Then the main trunk of the nerve goes around the brachial joint and enters the lateral surface of the scapula, where it innervates the deltoid muscle and the teres minor muscle. The suprascapular nerve is a strong nerve, which is mainly formed by branch C5. Sometimes it is also formed by a thin ramus of branch C4. It innervates the supraspinatus and infraspinatus muscles. The subscapular nerves are formed by branches C5 and C6 and they serve the median and cranial part of the subscapularis muscle. The lateral thoracic nerve is formed by branches C8 and T1. It goes to the chest wall together with the long thoracic nerve, which is formed by branches C7 and C8. The lateral thoracic nerve serves the cutaneus trunci muscle, whereas the long thoracic nerve serves the serratus ventralis muscle (Fig. 4). The fibres of branches C6-T1 form a strong nerve for the latissimus dorsi muscle, which is known as the thoracodorsal nerve. There are two cranial pectoral nerves formed by branches C6 and C7. They run towards the superficial and deep pectoral muscles (Fig. 5). The caudal pectoral nerve, which is formed from nerves C7-T1 (80%) or C8-T1 (20%), runs to the deep pectoral muscle.

## Discussion

The brachial plexus of the Djungarian hamster is mainly composed of the similar ventral branches of the spinal nerves as in the other members of the Muroidea superfamily described in scientific publications, e.g. the house mouse (*Mus musculus*) (Bogusch 1987) and lesser mole-rat (*Spalax leucodon*) (Aydin and Karan 2012) (Tab.1). The brachial plexus of the Djungarian hamster differs in three branches from the brachial plexus of the chinchilla (*Chinchilla lanigera*), which, according to Gamba et al. (2007), consists of branches C6-T1. The brachial plexus of the Djungarian hamster is different from the brachial plexus of the albino rat (*Mus norvegicus albinus*), a representative of the same superfamily (Muroidea), in which, according to Greene (1963), the plexus is formed from ventral branches C4-T1. A different range of ventral branches forming the brachial plexus of the rat was described by Uzun et al. (2001). The researchers used microscopic techniques in their study and noted that the plexus was formed by branches C5-T1, whereas C4 could only be included in the brachial plexus via the C5 nerve. Such a connection of the ventral branches can also be found in the Djungarian hamster, whose ventral branch of the C4 nerve becomes part of the brachial plexus via the C5 nerve. The brachial plexus of the Mongolian gerbil (*Meriones unguiculatus*) of the Muridae family is formed by nerves C4-T1 (Araujo et al. 2018). The brachial plexus of the Djungarian hamster is distinct from the plexuses of representatives of other rodent families. The brachial plexus of the red squirrel (*Sciurus vulgaris*), a representative of the Sciuromorpha family, is composed of four nerves (C5-C8) (Aydin 2011). Representatives of the Hystricognathi infraorder, which includes a large group of mammals, mostly differ considerably in the structure of their brachial plexuses. The brachial plexus of the nutria (*Myocastor coypus*) is composed of nerves C6-T1 (Guimaraes et al. 2013). Fioretto et al. (2003) found that the brachial plexus of the capybara (*Hydrochaeris hidrochaeris*) was composed of nerve branches C4-T1. The brachial plexus of the crested porcupine (*Hystrix cristata*) is composed of branches C5-T1 (Aydin 2003). Rodents are characterised by a wide range of spinal nerves in the brachial plexus. Their plexus structure is very similar to that of primates. Ribeiro et al. (2005) found that the brachial plexus of the tufted capuchin (*Sajapus apella*) was formed by branches C4-T2. The brachial plexus of the vervet (*Chlorocebus pygerythrus*), which is also an ape, is composed of branches C5-T2 (Booth 1991). There is high similarity between the branches of the human brachial plexus and those of the Djungarian hamster. In the classic variant the plexus is composed of branches C5-T1. However, it can extend between branches C4 and T2, which makes it a prefixed (C4-T1) or postfixed (C5-T2) plexus in some cases. Uysal et al. (2003) examined 200 human brachial plexuses and found that only 0.5% of them had both the C4 and T2 nerves at the same time. The brachial plexus can also be classified according to the number of trunks formed by the intertwining ventral branches of the spinal nerves. According to Aydin and Karan (2012), in the brachial plexus of the lesser mole-rat one trunk is formed by all branches. The brachial plexus of the red squirrel (*Sciurus vulgaris*) is composed of two trunks: cranial and caudal (Aydin 2011). There are three trunks in the human brachial plexus: superior, middle, and inferior (Uysal et al. 2003). The brachial plexus of rats is also composed of three trunks (Uzun et al. 2001). The radial nerve is the largest of the nerves emerging from the trunks in the brachial plexus of the Djungarian hamster – it is composed of branches C7, C8, and T1. Araujo et al. (2016) also noted that the radial nerve was the largest in the brachial plexus of the Spix’s yellow-toothed cavy (*Galea spixii*) – it was composed of branches C8, T1, and T2. The radial nerve of the capybara is composed of the same branches as that of the Djungarian hamster (C7-T1) (Fioretto et al. 2003). The radial nerve in the hamster is the main nerve responsible for innervating the extensors of the forearm and the skin on the lateral side of the forearm. A similar area of supply in nutria is reported by Guimares et al. (2013) however, it is reported that, the radial nerve is also involved in the innervation of the teres major muscle, which is not the case in the Djungarian hamster. Another strong nerve is the suprascapular nerve, which regularly innervates the supraspinatus and infraspinatus muscles. In the nutria this nerve derives from C5 and C6, but innervates the same muscles (Taketani 2017). The median nerve and the ulnar nerve, which are connected to each other in the Djungarian hamster, derive from C6-T1. Similarly, the median nerve of the Gambian pouched rat (*Cricetomys gambianus*) is formed from nerves C6-T1 and the ulnar nerve is formed from C7-T1 (Ibe et al. 2020), however, the author states that the median nerve alone innervates the deep digital flexor muscle and in the case of Djungarian hamster the median and ulnar nerves together innervate this muscle. Dual innervation of this muscle is also characteristic for humans. The T2 nerve may participate in the formation of the ulnar nerve of the Djungarian hamster, but not of the Mongolian gerbil (Araujo et al. 2018). The median nerve receives a connecting branch from the musculocutaneous nerve, which is also characteristic of howler monkeys (*Alouatta*). Such a branch was noted by Souza et al. (2018) in their study on the brachial plexus of the brown howler (*Alouatta guariba*), where the musculocutaneous nerve derived from nerves C5-C6 or C5-C7. Both the musculocutaneous nerve and axillary nerve of the Djungarian hamster derive from the C6 nerve. However, C7 is the common nerve of the Gambian pouched rat (*Cricetomys gambianus*) (Ibe et al. 2020). According to Green (1963), the axillary nerve of the rat is formed by branches C6 and C7. It is different from the range of this nerve in the Djungarian hamster, where there is a connection between branches C5 and C6, on which subscapular nerves are formed. Similarly, the subscapular nerves of the nutria are also formed as a result of the fusion of branches C5 and C6 (Taketani 2017). The cranial and caudal pectoral nerves innervate two layers of pectoral muscles. Both of these nerves range between branches C6 and T1 in the Djungarian hamster. However, both of them may derive simultaneously from the C7 branch. The cranial and caudal pectoral nerves of the brown howler also have fibres from the C7 branch (Souza et al. 2018). The long thoracic nerve of the Djungarian hamster is formed from branches C7-C8. However, this nerve is absent from the nutria (Guimaraes et al. 2013). Together with the long thoracic nerve, the lateral thoracic nerve, which innervates the cutaneus trunci muscle, spreads to the chest. The same situation was observed in the Spix’s yellow-toothed cavy (Araujo et al. 2016). Like in other rodents, the thoracodorsal nerve of the Djungarian hamster innervates the latissimus dorsi muscle (Araujo et al. 2016; Cevik-Demirkan et al. 2007). When peripheral nerves originating from the brachial plexus are damaged, there is impairment of the extensors and flexors of the forearm (Anderson et al. 1970; Fullerton and Gilliat 1967). Brachial plexus nerves at the level of the elbow joint are also damaged, resulting in paraesthesia, dysesthesia and even median nerve palsies (Lee and LaStayo 2004). As Vymazalová et al. (2015) points out, an additional predisposing factor for the aforementioned symptoms is the presence of a supracondylar foramen in the course of the median nerve, as in the Djungarian hamster. Some authors indicate a degenerative effect of mechanical factors on rodent nerves, which in this case refers to the effect of the pronator teres muscle on the median nerve (Ochoa and Marotte 1973; Vymazalová et al. 2015). In the clinical field, knowledge of brachial plexus anatomy is used during anaesthetic procedures. These are often used for brachial plexus anaesthesia (Bazin et al. 1997; Cao and Ling 2003; d’Ovidio and Adami 2019). Local anaesthesia of the plexus appears to be safer for the patient especially in light of studies showing fatalities with general anaesthesia in small animals (Brodbelt et al., 2008). In addition, it is worth noting that the key to developing surgical techniques, in human medicine, is progress and new information in animal model studies (Irintchev, 2011). To sum up, like in humans, the brachial plexus of the Djungarian hamster has three trunks. Similarly to other mammals of this order, the brachial plexus of the Djungarian hamster ranges widely (C5-T1). However, its nerves are formed from different ventral branches of the spinal nerves than in other mammals. Because of its structural similarity to the human and primate brachial plexus and similar innervation of the limb, it may be useful as a model for research on the human brachial plexus.

## Data Availability

The cadavers were delivered from veterinary clinics.

## Abbreviations

C4: ventral branch of C4
C5: ventral branch of C5
C6: ventral branch of C6
C7: ventral branch of C7
C8: ventral branch of C8
T1: ventral branch of T1
T2: ventral branch of T2
